# Age-Related Long-Term Functional Results after Riboflavin UV A Corneal Cross-Linking

**DOI:** 10.1155/2011/608041

**Published:** 2011-08-04

**Authors:** Aldo Caporossi, Cosimo Mazzotta, Stefano Baiocchi, Tomaso Caporossi, Rosario Denaro

**Affiliations:** ^1^Department of Ophthalmology, University of Siena, 53100 Siena, Italy; ^2^Department of Ophthalmology, Catholic University, Rome, Italy

## Abstract

*Purpose*. To report a comparative prospective long-term functional analysis after Riboflavin UV A corneal cross-linking (CXL) in three different age groups of patients affected by progressive keratoconus (KC). *Methods*. Functional analysis comprised paediatric patients (≤18 years) included 152 eyes (29.5%); intermediate group (19–26 years) 286 eyes (55.4%), and adults (≥27 years) 78 eyes (15.1%). CXL was performed according to the Siena protocol by using the Vega CBM (Caporossi-Baiocchi-Mazzotta) X linker (CSO, Florence, Italy) at Siena University by the same authors. Pre- and post-op examinations included UCVA, BSCVA, corneal topography, and surface aberrometry (CSO Eye Top, Florence, Italy), at 48 months followup. *Results*. At 48 months followup paediatrics, intermediate, and adult patients showed a mean gain in UCVA of +0.2, +0.14 and +0.12 Snellen lines. BSCVA gained by a mean of +0.21, +0.2, and +0.1 Snellen lines. *K*_max_ was reduced by a mean value of −0.9 D, −0.6 D, and −0.5 D, respectively. Coma values improved by a mean of −0.45 *μ*m, −0.91 *μ*m, and −0.19 *μ*m, respectively. Treatment ensured a long-term keratoconus stabilization in over 90% of treated patients. *Conclusion*. According to our long-term comparative results, epithelium-off Riboflavin UV A cross-linking should be the first choice therapy of progressive KC, particularly in paediatric age and patients under 26 years.

## 1. Introduction

Paediatric age at the time of diagnosis represents a negative prognostic factor for keratoconus progression, with increased probability of corneal transplant [1].

Particularly younger patients represent a population at high risk for more rapid progression of the disease [1, 2]. The long-term results reported in literature [3–5] have demonstrated the ability of cross-linking to slow the progression of keratoconus by a photo-polymerization reaction of stromal collagen fibres. Cross-linking photodynamic reaction is induced by the combined action of a photosensitizing substance (Riboflavina o vitamin B2) and ultraviolet (UV) A light, allowing a corneal stiffening by increasing the number of intrafibrillar, interfibrillar covalent bonds, and corneal collagen resistance against enzymatic degradation [6–8]. The long-term effects of the technique are related also to a process of collagen neosynthesis with a different structure and higher molecular weight [9–12] which confers to the corneal stroma an increased resistance and lamellar compaction responsible of the variable functional modifications recorded after the treatment [11–14]. 

According to international results [3–5], cross-linking should be the primary choice in young patient with progressive keratoconus.

## 2. Purpose

To report a comparative prospective long-term functional analysis after cross-linking in three different age groups (≤18 years, between 19–26 years, and ≥27 years) of patients affected by progressive keratoconus.

## 3. Methods

Since 2004 to date more than 610 patients were treated by combined Riboflavin UV A corneal collagen cross-linking. Present prospective nonrandomized open study comprised 516 eyes of 413 patients aged between 10 and 40 years, affected by progressive keratoconus. 

The comparative functional analysis comprised the following:

1. paediatric group (18 years and under) included 152 eyes of 105 patients (29.5%),
2. intermediate group (19–26 years) included 286 eyes of 243 patients (55.4%),
3. Adult group (≥27 years) included 78 eyes of 65 patients (15.1%).

### 3.1. Inclusion Criteria

All patients included in the treatment protocol were affected by progressive keratoconus with a documented clinical and instrumental worsening at least in the last three months of observation. The parameters we considered to establish keratoconus progression were worsening of UCVA/BSCVA > 0.50 Snellen lines, increase of SPH/CYL > 0.50 D, increase of topographic symmetry index SAI/SI > 0.50 D, increase of maximum *K* reading > 0.50 D, reduction of the thinnest point at optical pachometry ≥10 *μ*m, clear cornea at biomicroscopic examination, absence of reticular dark striae at confocal laser microscopy in vivo. Patients without possibility of optical correction were also included. We considered “significant” for the inclusion in the study the variation of at least 3 of the parameters listed above (one clinical plus two instrumental). Statistical analysis was conducted by the Mann-Whitney *U* test for nonparametric data (UCVA and BSCVA) and by the paired *t* test for parametric data (maximum curvature power, symmetry indices and coma values).

### 3.2. Surgical Technique

The surgical procedure of corneal cross-linking with Riboflavina UVA was performed in all patients according to the Siena protocol [3] using the Vega CBM (Caporossi-Baiocchi-Mazzotta) X linker (CSO, Florence, Italy) developed in Italy at the Department of Ophthalmology of Siena University by the same Authors, under intellectual property of Siena University, Italy. The treatment was conducted under topical anaesthesia (4% lidocaine drops). After applying the eyelid speculum, a 9 mm diameter marker was used to mark the corneal epithelium in a central circle, then epithelium was removed with a blunt metal spatula. After epithelial scraping, a disposable solution of Riboflavin 0.1% and Dextrane 20% (Ricrolin Sooft, Montegiorgio, Italy) was instilled for 10 minutes [15] of corneal soaking before starting UV A irradiation. The Riboflavin and Dextrane solution was administered every 2.5 minutes for a total of 30 minutes of UVA exposure (3 mW/cm^2^). Treated eyes were dressed with a therapeutic soft contact lens bandage for 4 days and medicated with antibiotics (Ofloxacin drops 4 times/day), nonsteroidal anti-inflammatory drugs (Diclofenac drops 4 times/day) and lachrymal substitutes, until contact lens removal. After therapeutic corneal lens removal, fluorometholone 0.2% drops (3 times/day) and lacrimal substitutes were administered for 6 to 8 weeks.

### 3.3. Followup and Assessment Criteria

(1) 152 eyes of 105 patients ≤18 years (91 eyes with followup of 12 months, 74 eyes at 24 months, 25 eyes at 36 months, 7 at 48 months).

(2) 286 eyes of 243 patients between 19 and 26 years (108 eyes with followup 12 months, 83 eyes at 24 months, 56 eyes at 36 months, 11 at 48 months).

(3) 78 eyes of 65 patients ≥27 (35 eyes with followup 12 months, 25 eyes at 24 months, 12 eyes at 36 months, 8 at 48 months).

Pre- and postoperative examination included uncorrected visual acuity (UCVA), best spectacle corrected visual acuity (BSCVA), corneal topography and surface aberrometry (CSO Eye Top, Florence, Italy), optical pachometry (Visante OCT, Zeiss Meditech, Jena, Germany), and in vivo confocal microscopy (HRT II, Heidelberg, Rostock Cornea Module, Germany).

## 4. Results

According to epidemiology findings [16], we found a male/female ratio in the whole sample of 4 : 1; a male/female ratio in paediatric group of 6 : 1, and a male/female ratio of 3 : 1 in patients between 19 and 40 years old. These results, even if in a small group, differ from epidemiological data reported in the literature [16] were a male/female rate of 2 : 1 is reported. There was no statistical difference in the incidence of keratoconus between right and left eye in the whole population of 516 eyes and age-related groups.

### 4.1. Functional Results

Comparative UCVA in patients ≤18 years showed a mean gain of +0.14 (*P* = 0.0037), +0.17 (*P* = 0.0043), +0.16 (*P* = 0.0051) and +0.2 (*P* = 0.006) Snellen lines at 12, 24, 36, and 48 months of followup, respectively. 

Patients between 19 and 26 years showed a mean gain of +0.13 (*P* = 0.0034), +0.16 (*P* = 0.0041), +0.12 (*P* = 0.0032), and +0.14 (*P* = 0.0073) Snellen lines at 12, 24, 36, and 48 months of followup, respectively. 

**Figure 1:**
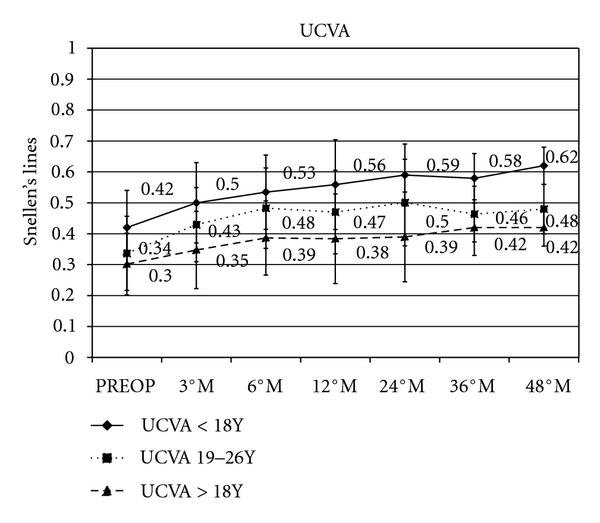
Graph showing visual performance during followup. After cross-linking, uncorrected visual acuity (UCVA) in patients ≤18 years showed a mean gain of +0.14, +0.17, +0.16, and +0.2 Snellen lines at 12, 24, 36, and 48 months of follow-up, respectively; in patients between 19 and 26 years showed a mean gain of +0.13, +0.16, +0.12, and +0.14 Snellen lines at 12, 24, 36, and 48 months of followup, respectively; and in patients ≥27 years showed a mean gain of +0.08, +0.09, +0.12, and +0.12 Snellen lines at 12, 24, 36, and 48 months followup, respectively.

Patients ≥27 years showed a mean gain of +0.08 (*P* = 0.0036), +0.09 (*P* = 0.005), +0.12 (*P* = 0.0047) and +0.12 (*P* = 0.0071) Snellen lines at 12, 24, 36, and 48 months of followup, respectively, (Figure 1). 

Comparative BSCVA in patients ≤18 years showed a mean gain of +0.15 (*P* = 0.0056), +0.19 (*P* = 0.0031), +0.18 (*P* = 0.0059) and +0.21 (*P* = 0.0079) Snellen lines at 12, 24, 36, and 48 months of followup, respectively. 

Patients between 19 and 26 years showed a mean gain of +0.10 (*P* = 0.0052), +0.12 (*P* = 0.0045), +0.13 (*P* = 0.0056), and +0.2 (*P* = 0.0075) Snellen lines at 12, 24, 36, and 48 months of followup, respectively. 

**Figure 2:**
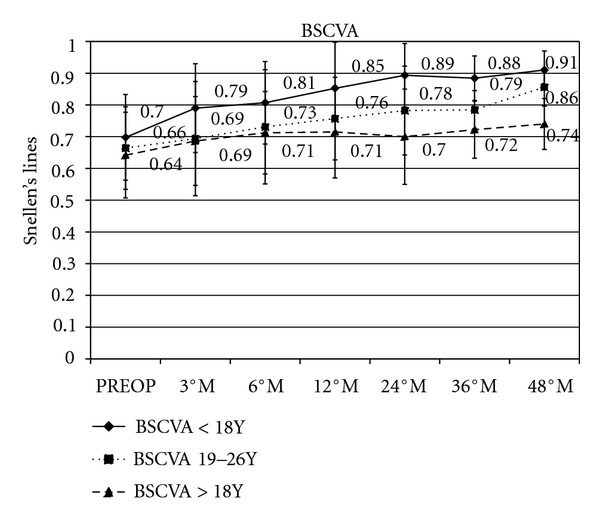
Graph showing visual performance during followup. After cross-linking, best spectacle-corrected visual acuity (BSCVA) in patients ≤18 years showed a mean gain of +0.15, +0.19, +0.18 and +0.21 Snellen lines at 12, 24, 36, and 48 months of followup, respectively; in patients between 19 and 26 years showed a mean gain of +0.10, + 0.12, +0.13, and +0.2 Snellen lines at 12, 24, 36, and 48 months followup, respectively; in patients ≥27 years showed a mean gain of +0.07, +0.06, +0.08, and +0.10 Snellen lines at 12, 24, 36 and 48 months of followup, respectively.

Patients ≥27 years showed a mean gain of +0.07 (*P* = 0.0054), +0.06 (*P* = 0.0067), +0.08 (*P* = 0.0069), and +0.10 (*P* = 0.0075) Snellen lines at 12, 24, 36, and 48 months of followup, respectively, (Figure 2).

### 4.2. Topography Results

**Figure 3:**
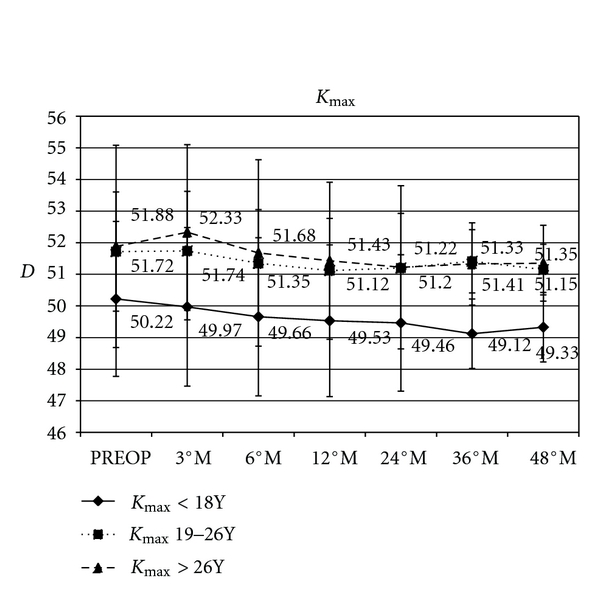
The graph showing computerized corneal topography results. The comparative topographic analysis showed a *K*_max _ variation in paediatric group by a mean of −0.7 D, −0.8 D, −1.1 D, and −0.9 D; in the intermediate group (patients between 19 and 26 years) varied by a mean of −0.6 D, −0.5 D, −0.3 D, and −0.6 D; in adult group (patients ≥27 years) varied by a mean of −0.4 D, −0.6 D, −0.5 D, and −0.5 D, at 12, 24, 36, and 48 months of followup, respectively, for each group.

*K*_max_ in paediatric group varied by a mean of −0.7 D (*P* = 0.006), −0.8 D (*P* = 0.0045), −1.1 D (*P* = 0.051), and −0.9 D (*P* = 0.071); in the intermediate group (patients between 19 and 26 years) varied by a mean of −0.6 D (*P* = 0.0053), −0.5 D (*P* = 0.0051), −0.3 D (*P* = 0.0045), and −0.6 D (*P* = 0.0091); in adult group (patients ≥27 years) varied by a mean of −0.4 D (*P* = 0.0065), −0.6 D (*P* = 0.0074), −0.5 D (*P* = 0.0095), and −0.5 D (*P* = 0.0091), at 12, 24, 36, and 48 months of follow-up, respectively for each group (Figure 3). 

**Figure 4:**
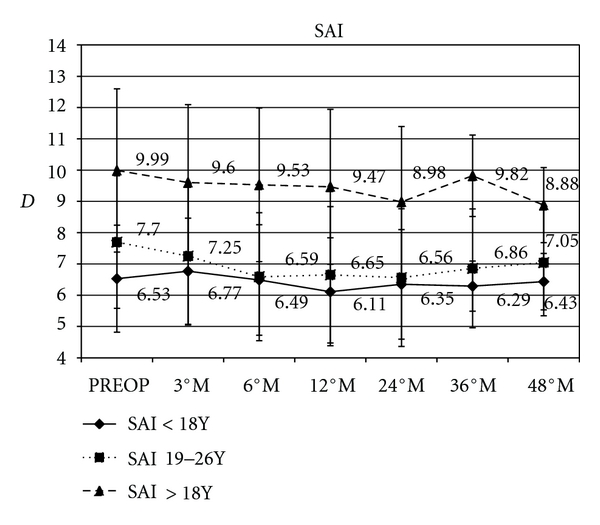
Graph showing anterior corneal surface aberrometry after corneal cross-linking (SAI m) in paediatric patients improved by a mean of, −0.42 D, −0.18 D, −0.24 D, and −0.10 D; in the intermediate group improved by a mean of, −1.05 D, −1.14 D, −0.84 D, and −0.65 D; in adult group improved by a mean of, −0.52 D, −1.0 D, −0.17 D, and −1.11 D.

Surface asymmetry index (SAI m) in paediatric patients improved by a mean value of, −0.42 D (*P* = 0.0054), −0.18 D (*P* = 0.0066), −0.24 D (*P* = 0.091), and − 0.10 D (*P* = 0.096); in the intermediate group improved by a mean of, −1.05 D (*P* = 0.0032), −1.14 D (*P* = 0.0021), −0.84 D (*P* = 0.0036), and −0.65 D (*P* = 0.076); in adult group improved by a mean of, −0.52 D (*P* = 0.0067), −1.0 D (*P* = 0.0077), −0.17 D (*P* = 0.0081), and − 1.11 D (*P* = 0.0094), (Figure 4). 

**Figure 5:**
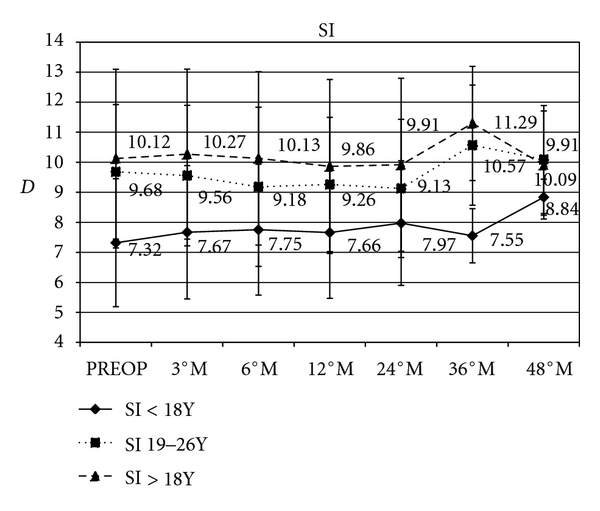
Graph showing topographic analysis of corneal symmetry using the superior-inferior index (SI). After cross-linking SI improved in paediatric patients by a mean of +0.3 D, +0.6 D, +0.2 D and +1.5 D; in the intermediate group changed by a mean of: −0.42 D, −0.55 D, +0.90 D, +0.40 D; in adult patients varied by a mean of: −0.26 D, −0.21 D, +1.17 D, −0.21 D at 12, 24, 36 and 48 months of follow-up respectively.

Topographic superior-inferior symmetry index (SI m) in paediatric patients varied by a mean of, +0.3 D (*P* = 0.0098),+ 0.6 D (*P* = 0.011), +0.2 D (*P* = 0.017), and +1.5  D (*P* = 0.021); in the intermediate group changed by a mean of, −0.42 D (*P* = 0.0086), −0.55 D (*P* = 0.0079), +0.90 D (*P* = 0.091), and +0.40 D (*P* = 0.099); in adult patients varied by a mean of, −0.26 D (*P* = 0.0059), −0.21 D (*P* = 0.0048), +1.17 D (*P* = 0.012), and −0.21 D (*P* = 0.0011) at 12, 24, 36, and 48 months of followup, respectively, (Figure 5).

### 4.3. Aberrometry Results

**Figure 6:**
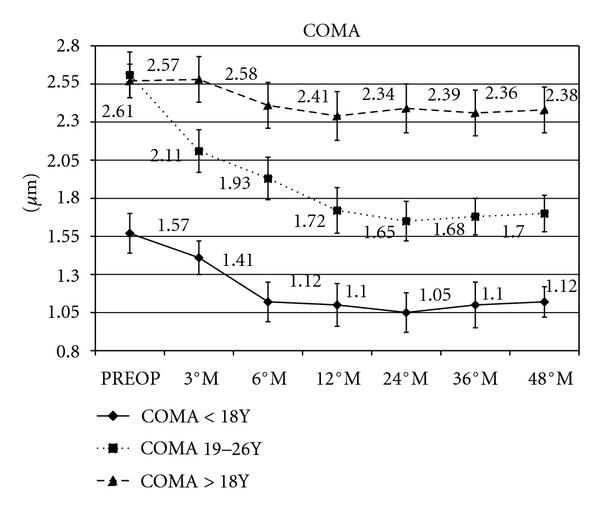
Graph showing the COMA analysis and the improving value in paediatric patients by a mean of −0.47 *μ*m, −0.52 *μ*m, −0.47 *μ*m, and −0.45 *μ*m; in the intermediate group coma values decreased by a mean of −0.89 *μ*m, −0.96 *μ*m, −0.93 *μ*m, and −0.91 *μ*m; in old patients we recorded a mean postoperative value of −0.2 *μ*m, −0.18 *μ*m, −0.21 *μ*m, and −0.19 *μ*m at 12, 24, 36, and 48 months of followup, respectively.

Coma values in paediatric patients improved by a mean of −0.47 *μ*m (*P* = 0.0034), −0.52 *μ*m (*P* = 0.0025), −0.47 *μ*m (*P* = 0.0022), and −0.45 *μ*m (*P* = 0.0054); in the intermediate group coma values decreased by a mean of −0.89 *μ*m (*P* = 0.0034), −0.96 *μ*m (*P* = 0.0065), −0.93 *μ*m (*P* = 0.0074), and −0.91 *μ*m (*P* = 0.0081); in old patients we recorded a mean postoperative value of −0.2 *μ*m (*P* = 0.0056), −0.18 *μ*m (*P* = 0.0045), −0.21 *μ*m (*P* = 0.0034), and −0.19 *μ*m (*P* = 0.0067) at 12, 24, 36 and 48 months of followup respectively, (Figure 6).

## 5. Discussion

According to the literature in [1] and our previous reports [3], keratoconus progression is more frequent and faster in younger patients under 18 years old at the time of diagnoses, with higher probability to undergo a corneal transplantation [1, 3]. Therefore paediatric patients represent the goal of photo-induced Riboflavin UV A corneal collagen cross-linking [3]. 

The Italian pilot study “Siena CXL Paediatrics”, conducted on a large cohort of patients with a long-term follow up, demonstrated that there was a significant and fast functional improvement in younger patients after Riboflavin UV A corneal cross-linking.

As we recently published, it is however impossible to exactly predict the distribution of cross-links and the geometric redistribution of newly formed collagen [12, 14]. 

Long-term comparative analysis showed that functional results after Riboflavin UV A corneal collagen cross-linking among paediatric patients were slightly better, but without statistically significant differences with the results recorded in the intermediate group patients. On the other hand, patients over 27 years showed a positive but poorer functional response compared with other age groups. 

The mean *K*_max _ variation and topographic surface asymmetry index results were statistically significant in the paediatric sample, particularly in the postoperative 24 months. After the 24th month, until the 48th month, the mean data results were statistically not significant, reasonably due to reduced number of patients in the longitudinal analysis. 

The comparative aberrometric data of coma values showed a significant improvement in all analyzed groups, justifying the rapid improvement of visual acuity in all treated patients. In the paediatric group of our cohort, there was a minority of patients (about 5%) that, despite the treatment, showed a worsening trend or at least an instability of keratoconus. In our opinion, this concept should be remarked because the disease in this age group is more aggressive and the possibility of progression higher than in the others age groups. The instability of certain cases should be explained by the different genetic patterns of keratoconus [17–19] with relative biochemical modifications [20] potentially occurring in corneal stroma associated with negative influences of some environmental factors (allergy, atopy) [21–24]. 

Every time we decide to treat a paediatric patient under 18 years affected by progressive keratoconus, parents and patient himself should be well informed about the possibility that the treatment in a minority of cases could not warrant a total and long-lasting stabilization of the disease, with the possibility to repeat the cross-linking or to undergo alternative treatments. The “Siena CXL Paediatrics” pilot study demonstrated the effective ability of corneal cross-linking to retard keratoconus progression in all age groups with better functional response in patients under 26 years. Treatment ensured a long-term keratoconus stabilization in over 90% of treated cases. The lower functional response observed in patients over 27 years may be explained by a reduced collagen “plasticity” in the adult age, as well demonstrated in the literature [25, 26]. Cross-linking treatment may result in less effectiveness with increased failure and complication rate particularly in adult patients over 35 years, as reported in the literature [21]. According to our long-term comparative age-related analysis and results, the standard Riboflavin UV A cross-linking with epithelium removal should be the first choice therapy of progressive keratoconus in paediatric and under-26-year old patients with corneal thickness at least of 400 *μ*m in the thinnest point.
